# *Francisella tularensis* Bacteremia: A Case Report from Sudan

**DOI:** 10.1155/2012/405737

**Published:** 2012-07-09

**Authors:** Salma E. R. Mohamed, Aymun I. Mubarak, Lamia O. Alfarooq

**Affiliations:** ^1^Microbiology Laboratory, Sudan Peritoneal Dialysis Program, National Ribat University Center, P.O. Box 11111, 35 Burri, Khartoum, Sudan; ^2^Department of Medicine, Sudan Peritoneal Dialysis Program, National Ribat University Center, P.O. Box 11111, 35 Burri, Khartoum, Sudan

## Abstract

*Francisella tularensis* is a highly virulent intracellular gram-negative bacterium. The organism is usually isolated from wild and domestic animals and invertebrate. Man gets infection by direct contact with those animals or their products but the most common mode of transmission is via arthropod vectors. The disease is endemic in North America, parts of Europe, and Asia but has never been reported in Africa. A 29-year old male living in a rural area of Southern Sudan has been maintained on continuous ambulatory peritoneal dialysis for two years. He presented to our center in May 2010 complaining of fever, dry cough, shortness of breath, and abdominal discomfort for four days. He was very ill, pale, and dehydrated. There were enlarged tender submandibular lymph nodes, but no mouth ulcers or other palpable lymph nodes. Peritonitis was excluded by effluent white blood cell count and culture. Empiric antibiotic treatment with ceftriaxon, and ciprofloxacin was started. Gram-negative coccobacilli were isolated by blood culture. The organism was identified as *Francisella tularensis*. We started him on a ten-day course of gentamicin after which he improved. This is, to the best of our knowledge, the first reported case of bacteremia caused by *Francisella tularensis* in Sudan.

## 1. Introduction

*Francisella Tularensis *is a highly virulent intracellular gram-negative bacterium [1]. The organism is usually isolated from wild and domestic animals and invertebrates. Humans get infection by direct contact with those animals or their products, but the most common mode of transmission is via arthropod vectors; ticks and mosquitoes. The disease is endemic in North America, parts of Europe, and Asia but has never been reported in Africa [2]. This may be due to its low incidence, misdiagnosis, or inadequate laboratory facilities and expertise.

## 2. Case Report

A 29-year old male living in a rural area of Southern Sudan has been maintained on continuous ambulatory peritoneal dialysis for two years. He presented to our center in May 2010 complaining of fever, dry cough, shortness of breath, and abdominal discomfort of four days duration. He was very ill, pale, and dehydrated. His temperature was 38.4°C, respiratory rate 30 per min, pulse rate 120 beats per minute, and blood pressure 70/50 mmHg. There were enlarged tender submandibular lymph nodes, but no mouth ulcers or other palpable lymph nodes. Chest and precordium examination was unremarkable. Abdominal examination revealed bilateral loins tenderness but no guarding. There was no palpable organomegaly, and the peritoneal catheter exit site showed no signs of inflammation. The skin was intact and there was no arthritis. Neurological examination revealed no abnormality.

The patient was admitted and rehydrated. The peritoneal effluent was inspected and found to be clear. Investigations revealed WBCs count of 13,100/*μ*L with 90% polymorphonuclear cells, hemoglobin 5.6 g/dL, platelets 160,000/*μ*L, ESR > 150 mm/hr, serum albumin 1.9 mg/dL, blood urea 168 mg/dL, serum creatinine 9.2 mg/dL, and normal liver enzymes and electrolyte levels. Peritoneal effluent contained 50 WBC/*μ*L. Blood film for malaria was negative as were serological tests for Brucella and Salmonella.

We sent blood and peritoneal effluent samples for culture and started empiric antibiotic treatment with ceftriaxone, and ciprofloxacin. He also received 4 units of blood to correct his anemia.

Peritoneal effluent cultures remained negative after three days of incubation. Blood samples were cultured in Brain heart infusion (BHI) broth and Thioglycollate broth. After five days of incubation, the aerobic BHI broth showed increased turbidity and gram stain revealed tiny gram negative pleomorphic bacilli. Subcultures were performed in three solid media; blood agar, chocolate agar, and McConkey agar. Forty eight hours later the subcultures showed non hemolytic mucoid gray white colonies on the chocolate agar, very faint growth was detected on the blood agar, and no growth noticed on the McConkey agar plate.

Giemsa stain smear revealed a bipolar organism. The isolated organism tested negative for oxidase, urease, motility and fermentation of lactose, and other sugars. A battery of chemical tests was performed utilizing two analytical profile index kits (API, bioMerieux) for identification of gram-negative rods; API-20E and API-20NE. Both failed to identify the organism but confirmed that it did not belong to *hemophilus*, *actinobacillus*, *cardiobacterium*, *ekinella* or *kingella* species. Thus, the organism was identified as *Francisella tularensis*. Further investigations to determine antimicrobial sensitivity were not done due to inadequate lab safety facilities.

The patient's condition was generally better but he was still running on and off fever. We started him on a ten-days course of gentamicin. The patient improved dramatically after receiving gentamicin and was discharged in good condition. He remained asymptomatic two weeks later when he presented for followup. No similar condition emerged in his family or contacts including the medical and laboratory personnel. To the best of our knowledge, this is the first reported case of *Francisella* septicemia in our area and, may be, in Africa.

## 3. Discussion

*Francisella tularensis* is a facultative intracellular gram-negative bacterium, with four subspecies; *tularensis*, *plaearctica*, *mediasiatica*, and *novicida* [3]. Due to its ease of spread by aerosol it is considered to be a highly virulent organism [3].

*Francisella tularensis* infection can be acquired through the skin, eyes, mouth, throat, or lungs. Mosquitoes are considered the primary vector, but humans can be infected by direct contact with infected animals or following ingestion of contaminated water and food or inhalation of aerosolized bacteria. Human-to-human transmission is considered rare [1, 2].

Depending on the site of infection, tularemia has six characteristic clinical syndromes; ulceroglandular (the most common type representing 75% of all forms), glandular, oropharyngeal, pneumonic, oculoglandular, and typhoidal (bacteremia) [1].

Bacteremia caused by *Francisella tularensis* (tularemia) is a rare but potentially fatal condition. Symptoms of all forms of tularemia typically include fever with chills, headache, sore throat, generalized body aches, and malaise. Symptoms usually develop within 3–5 days of infection; however, the incubation period can vary from 1–14 days. Without prompt treatment, patient becomes severely toxic and may develop sepsis, septic shock, acute respiratory distress or multiorgan failure, then confusion and coma [1].

Firsts line treatment options include streptomycin, gentamicin, and tetracycline-class drugs, such as, doxycycline. Chloramphenicol and quinolones have also been successfully used for treatment [4, 5]. Besides microbiological isolation, the diagnosis can be confirmed by PCR or a four-folds increase in specific antibody titer [6].

In this case we could not reliably identify the source of infection. The patient had no history of travel and no direct contact with infected animal. However, he lived in a rural area and could have been exposed to mosquitoes, contaminated meat or water, or contaminated dust.

*Francisella tularensis* is endemic in some areas of the world; including North America, Europe, and northern Asia [2]. No cases were officially reported from Africa to the best of our knowledge. In the presented case, tularemia infection was not suspected initially. It was only considered after exclusion of the more common infectious diseases in our area. This case underlines the importance of considering this diagnosis in our part of the world.

## 4. Conclusion

This is, to the best of our knowledge, the first reported case of bacteremia caused by Francisella tularensis in Sudan.
